# Riding the Pandemic Waves—Lessons to Be Learned from the COVID-19 Crisis Management in Romania

**DOI:** 10.3390/tropicalmed7070122

**Published:** 2022-06-29

**Authors:** Gergő Túri, János Kassay, Attila Virág, Csaba Dózsa, Krisztián Horváth, László Lorenzovici

**Affiliations:** 1 Epidemiology and Surveillance Centre, Semmelweis University, 1085 Budapest, Hungary; 2 Faculty of Economics, Socio-Human Sciences and Engineering, Sapientia Hungarian University of Transylvania, 400112 Cluj-Napoca, Romania; kassayjanos@uni.sapientia.ro; 3 Institute for the Development of Enterprises, Corvinus University of Budapest, 1093 Budapest, Hungary; attila.virag@uni-corvinus.hu; 4 Department of Basic Health Sciences and Health Care Management, University of Miskolc, 3515 Miskolc, Hungary; csaba.dozsa69@gmail.com; 5 Department of Public Health, Semmelweis University, 1085 Budapest, Hungary; krisztian.horvath21@gmail.com; 6 Syreon Research Romania, 54004 Tirgu Mures, Romania; lorenzovici@syreon.ro; 7 George Emil Palade University of Medicine, Pharmacy, Science and Technology, 540139 Tirgu Mures, Romania

**Keywords:** Romania, health policy, COVID-19, pandemic management, pandemic response, surveillance

## Abstract

In our analysis, we assessed how Romania dealt with the numerous challenges presented by the COVID-19 pandemic during 2021. In that year, the government had to deal with two waves of COVID-19 pandemics caused by the new variants, the low vaccination rate of the population, the overload of the healthcare system and political instability at the same time. Based on publicly available databases and international literature, we evaluated government measures aimed at reducing the spread of the pandemic and ensure the operation of the healthcare workforce and infrastructure. In addition, we evaluated measures to provide health services effectively and the government’s pandemic responses regarding excess mortality in 2021. In the absence of a complex monitoring system, limited information was available on the spread of the pandemic or the various risk factors at play. Due to incomplete and inadequate management systems, the government was unable to implement timely and adequate measures. Our analysis concludes that the management of a pandemic can only be successful if data are collected and evaluated using complex systems in a timely manner, and if members of society adhere to clearly communicated government measures due to high levels of trust in the government.

## 1. Introduction

Romania is an Eastern European country with a population of approximately 19.5 million people. The World Bank first ranked the country as a high-income country in 2019, however, in 2020, due to the social and economic consequences of the COVID-19 pandemic, the organization reclassified Romania as a middle-income country [1]. In 2020, life expectancy at birth was 74.2 years in Romania, the second lowest in the European Union [2]. The healthcare system is funded by compulsory health insurance, with governmental health spending accounting for 5.7% of GDP in 2019, the second-lowest in the European Union. The first confirmed COVID-19 case in Romania was reported on 26 February 2020, and in 2020, there were two waves of pandemics in the country [3].

The year 2021 brought several challenges in the fight against the COVID-19 pandemic, which was responsible for 5.9 million deaths globally and 63,578 deaths in Romania up to 1 March 2022. An important new tool in the fight against the pandemic became available in the form of COVID-19 vaccines; however, the implementation of the vaccination programs has been a major challenge for health systems struggling with the pandemic nearly a year ago. Several new variants emerged causing several epidemic waves since the outbreak of the coronavirus in early 2020 [4]. Both the low vaccination willingness of the population and the decline in vaccine efficacy over time have emerged as major issues in several countries and need to be addressed [5,6].

Romania’s pandemic management has been hampered by factors such as low confidence in the government and the healthcare system’s ability, and a general state of inadequate healthcare infrastructure and lacking human resources [3]. Before the COVID-19 pandemic, the Romanian healthcare system was characterized by inadequately structured workforce capacity and hospital infrastructure not up to date to the requirements of contemporary healthcare needs of the population [7]. In 2020, more than half a million people lived in communities where there was no general practitioner [8]. Territorial inequalities also existed in the context of secondary care, with insufficient physicians in intensive care units in smaller and medium-sized hospitals. [9] Shortcomings in the training of public health experts and the low number of public health professionals posed extraordinary challenges to epidemiological surveillance at the start of the pandemic [10]. From March 2020, the burden on the healthcare system was exacerbated by the fact that all COVID-19 positive cases were compulsorily hospitalized, regardless of whether they had symptoms [11]. The court only lifted this regulation in August 2020.

Assessing responses to new and existing challenges is particularly important in the context of the COVID-19 pandemic. Areas for improvement need to be identified and good practices explored, that could contribute to better management of future pandemics caused by respiratory viruses. There are several frameworks available to assess a countries preparedness and response capabilities to a health crisis such as COVID-19. An important theoretical framework for assessing pandemic preparedness and response was the International Health Regulations (IHR) adopted by the World Health Assembly (WHA) in 2005 [12,13]. The purpose of the IHR was to support the preparation of WHA member countries for the prevention and identification of health emergencies and to facilitate their response to emergencies. The IHR has identified four key areas, such as (i) preventing the likelihood of outbreaks, (ii) detecting health threats, (iii) implementing rapid and multisectoral responses, and (iv) developing capacities at the point of entry. Based on this framework, several countries prepared a pandemic plan and a self-assessment for the World Health Organization [13,14].

From 2017, the pandemic responsiveness of some countries was also assessed with the involvement of independent experts. In the early stages of the COVID-19 pandemic, the WHO established the COVID-19 Strategic Preparedness and Response Plan Monitoring and Evaluation Framework (SPRMEF) [15]. The framework has supported the development of Member States’ resilience to the pandemic and has facilitated the comparison of country-specific measures [16]. The SPRMEF identified nine main pillars, including new elements compared to the IHR system, such as case management, operational support, and logistics, and maintaining essential health services during an outbreak.

The COVID-19 Health System Response Monitor (HSRM), a joint initiative of the European Observatory on Health Systems and Policies, and the WHO Regional Office for Europe, supports a structured analysis of pandemic management in a country and comparisons between countries [17]. The HSRM examines a country’s health policy responses to the COVID-19 pandemic along with the following five areas: preventing local transmission, ensuring sufficient physical infrastructure and workforce capacity, providing health services effectively, paying for services, governance, and measures in other sectors. Similar to several previous analyses evaluating the health policy responses of the COVID-19 pandemic in countries, we adapted the structure of HSRM for our analysis [18,19,20]. The HSRM framework was chosen because it focuses on critical areas of pandemic response, uses a broad set of criteria, and allows for the systematic organization of relevant information and data.

Our study aimed to evaluate the experiences and lessons learned from pandemic management in Romania in the year 2021. Using Romania as a case study, we highlight the pandemic management of a middle-income country in Eastern Europe struggling with several difficulties simultaneously, such as the low vaccination of the population and new waves of pandemics due to new COVID-19 variants. Our analysis has complemented research on pandemic management in low- and middle-income countries. Several countries have struggled to operate appropriate surveillance systems, develop the population’s willingness to comply with restrictions, and develop vaccination willingness [21,22,23,24]. However, several low- and middle-income countries also provided many good practices in managing the pandemic [25,26].

The results of our study can contribute to the improvement of pandemic management plans and actions in Romania and can also serve as a lesson for other low- to middle-income countries globally. The specific objectives were as follows:

1. To assess the implemented non-pharmaceutical measures aimed at reducing the pandemic’s spread and to evaluate implemented measures to ensure sufficient workforce capacity and infrastructure (Section 1 and Section 2 in HSRM profiles);
2. To describe the implemented government structure to manage the pandemic and to evaluate the implemented measures to provide health services effectively (Section 3 and Section 5 in HSRM profiles);
3. To evaluate the government’s pandemic responses in 2021 regarding excess mortality;
4. To ascertain the main experiences and lessons that can be learned from managing the pandemic in Romania.

## 2. Materials and Methods

The data collection timeframe for our analysis was 1 January 2021 to 31 December 2021. In order to identify the most important non-pharmaceutical measures in Romania between January and December 2021, we reviewed official governmental and international news sites. Non-pharmaceutical measures are actions other than vaccinations and medications that can help communities slow the spread of an epidemic. Data from the Our World in Data (OWID) and European Centre for Disease Control and Prevention (ECDC) databases were used to assess the implemented measures to counter the spread of the pandemic (Table 1) [27,28].

The stringency index developed by the University of Oxford was used to characterize the severity of the non-pharmaceutical measures applied in Romania, where a higher score indicates a stricter response. The stringency index consists of nine components: closing schools, closing jobs, postponing community events, restricting gatherings, public transport closure, stay-at-home measures, communication campaign, international travel restrictions, and international travel controls. The stringency index is calculated as the mean score of the nine metrics, each taking a value between 0 and 100. Each indicator reflects the most stringent government policy that is in place in a country, as represented by the highest ordinal value. If the most stringent policy is only present in a limited geographic area, then a binary flag variable is used to denote this limited scope. The stringency index does not include all non-pharmaceutical measures for a pandemic response but provides a comprehensive picture of the nature of the most significant measures. However, as the stringency index only shows the rigor of government measures and policies, not their effectiveness or timeliness, we examined additional epidemiological indicators, such as confirmed COVID-19 cases per million people and weekly test positivity rate. Information on measures to improve healthcare workforce capacity was gathered through a review of official government websites and reliable news sites. Data from the Eurostat database were used to describe healthcare workforce capacity and hospital infrastructure in Romania [29].

The description of the governmental structure for pandemic management was based on country profiles prepared by the HSRM and the OECD. The governmental structure is the institutional system responsible for managing the pandemic and its operating mechanism. A scoping literature review was carried out on the vaccination program in Romania, the factors influencing the population’s vaccination willingness, and the measures aimed to ensure the continued operation of the healthcare system. The scoping literature review was performed according to the guideline developed by the Joanna Briggs Institute. In the protocol, we formulated the areas of literature research, the method of the search strategy, the selection criteria, and the source of evidence selection. In addition, the methodology for data extraction, analysis, and presentation was defined in the protocol [30].

The literature search was performed on PubMed, Medline, and Google Scholar databases using the following keywords: Romania, COVID-19, vaccination, vaccination program, vaccination hesitancy, and vaccination willingness. The publication’s inclusion criteria were as follows: the publication should include information on the purpose and methods of the vaccination program in Romania, the factors influencing the vaccination willingness, and the measures aimed at the health system. Additional criteria were the availability of full texts, in the English or Romanian language. Exclusion criteria were publications more than two years old and publications that did not contain information on the causes of low vaccination willingness in Romania or information on the vaccination program and healthcare measures in Romania. The literature search resulted in 75 relevant articles. We screened the results first by their title, and the most relevant ones by their abstract, summarizing our findings based on seven articles.

Data from the OWID and ECDC databases were used to evaluate the provision of health services. The following indicators were examined: number of COVID-19 patients in ICU per million people and number of fully vaccinated people per hundred. In order to evaluate the Romanian government’s pandemic responses in 2021 regarding excess mortality, the following indicators were examined: weekly number of confirmed COVID-19 death per million people and weekly excess death per million people. Data from the OWID database were used in the examination. Based on the results of our analysis, we have formulated the experiences and lessons that can help improve further the management of the pandemic in Romania and the fight against pandemics in low- and middle-income countries facing similar challenges.

## 3. Results

### 3.1. Preventing Local Transmission

**Figure 1:**
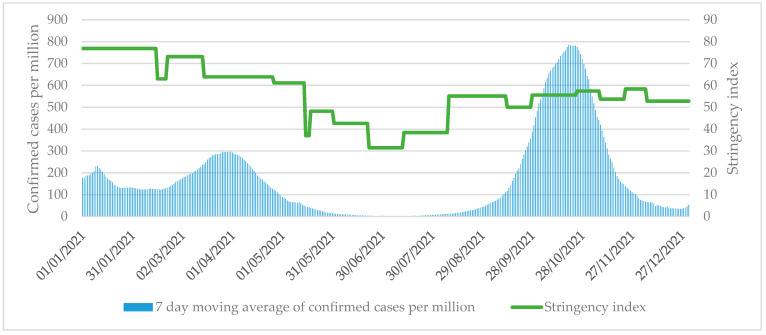
Stringency index values and confirmed COVID-19 cases per million in Romania between 1 January 2021 and 31 December. Source: University of Oxford [27].

Several indicators need to be examined simultaneously to determine whether the government has responded appropriately and on time when evaluating measures to prevent local transmission. There were two waves of pandemics in Romania in 2020, while in 2021, a third and fourth waves hit the country [31]. The third wave was from the beginning of February to the end of June 2021, and the fourth wave was from July to mid-December 2021. At the beginning of the third wave in February 2021, severe restrictions were still in place, but the stringency index value fell from 76 to 63 on 8 March (Figure 1). The curfew and restrictions on schools have been partially eased despite the resurgence of the epidemic from 12 February. At the peak of the third wave on 31 March, the 7-day moving average of confirmed cases per million population was 296.

Between 15 May and July, restrictions were eased on several occasions (Table 2). The stringency index rose during August, but it remained lower than before, hovering between 50 and 60 for the rest of the year, although the 7-day moving average of the confirmed cases at the peak of the fourth wave on 25 October was 2.66 times that of the third wave.

An important indicator of the testing strategy is the share of the COVID-19 tests that are positive. According to the WHO recommendation, the ideal is to keep the positivity rate below 5% [11]. However, a positivity rate higher than the WHO criteria means that the intensity of testing does not match the rate of spread of the pandemic, so the country’s surveillance system misses many cases. In Romania, there were only 24 weeks in 2021 when the positivity rate was below 5%, so testing capacity was adequate in less than half (46%) of the year (see Supplementary Figure S1.).

However, while only PCR tests were used in the initial phase of the pandemic, from November 2020, antigen rapid tests were increasingly used by government and private providers and the general public, thus expanding testing capacities [39]. The year 2021 also showed that virus sequencing programs have an important role in pandemic management, as they provide essential information about the characteristics of new variants and their prevalence [40]. The ECDC issued a representative and targeted genomic SARS-CoV-2 monitoring recommendation in May 2021 [41]. The ECDC recommends representative sampling and sequencing of cases identified during routine surveillance to identify and monitor new variants. In addition to the general methodological recommendations, the guidance includes country-specific recommendations in respective sample sizes and options for specimen selection. A properly implemented sequencing program can provide critical information for planning non-pharmaceutical measures by identifying a new variant and the rate of spread of the variant. According to the ECDC database, in the first half of 2021, Romania mainly reported an inadequate sequencing volume with insufficient precision at a variant prevalence of 5% (out of the first 26 weeks, only 4 weeks were adequate) (see Supplementary Table S1). However, Romania improved its sequencing capacity and reporting system in the second half of the year and reported an inadequate sequencing volume with insufficient precision for only 6 weeks.

### 3.2. Ensuring Sufficient Health Workforce Capacity and Infrastructure

The number of practicing medical doctors per hundred thousand inhabitants was below the EU average in 2019, and although Romania trained doctors and nurses above the EU average, the emigration of the healthcare workforce has been a severe problem for years (Table 3).

Although the number of hospital beds per 100,000 people in Romania was above the EU average, the intensive care unit was already short of workforce capacity in 2019 [2]. Workforce and infrastructure problems remained significant in the Romanian healthcare system in 2020 and 2021 as well [2]. In 2021, especially during the fourth wave of the epidemic, there was a shortage of medical oxygen and drugs in Romanian hospitals [42]. The European Commission and several EU member states provided oxygen concentrators and drugs in October 2021 in order to help ease the pressure mounting on the healthcare system. Nearly 2000 jobs were created for district public health centers and emergency departments, medical students were asked to volunteer, and redeployments within and between institutions were made as needed [17]. Financial incentives (salary increases, bonuses, housing benefits) and burnout prevention services have also been offered to help retain the healthcare workforce.

### 3.3. Providing Health Services Effectively

**Figure 2:**
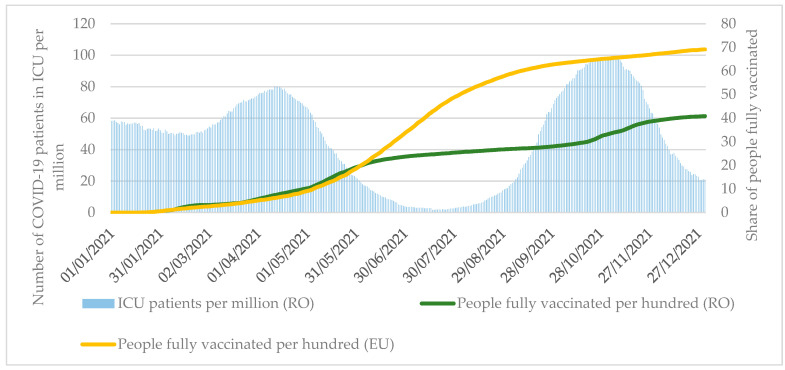
Number of COVID-19 patients in ICU per million and number of fully vaccinated people per hundred in Romania between 1 January and 31 December 2021. Source: University of Oxford [21].

In 2021, one of the critical services of the healthcare system was the proper design and implementation of the COVID-19 vaccination program. As Romania joined the Joint Procurement Agreement coordinated by the European Union, it was able to obtain vaccines through this mechanism, in addition to medical devices, services, and medicines [43]. In the first half of the year, the immunization of the Romanian population progressed at the same rate as the EU average. By 19 June, 19.6% of the Romanian population had been fully vaccinated, in line with the EU average (Figure 2). Fully vaccinated refers to having received two doses of the Pfizer-BioNTech/Moderna/AstraZeneca vaccines or one dose of the Janssen vaccine.

The vaccination program in Romania slowed down in the summer compared to the EU average, with 26.8% of the Romanian population vaccinated by the end of August. At that time, only 34.3% of those over 60, a particularly vulnerable group, were fully vaccinated, compared to the EU average of 82.9% (see Supplementary Table S2). The vaccination program was only able to accelerate before the peak of the fourth wave of the pandemic, but it was less able to influence the health losses of the pandemic. Only 40.8% of the Romanian population was fully vaccinated by the end of 2021, compared to the EU average of 69.1%. Among those over 60, vaccination rates remained particularly low (45.5%) than the EU average (89.1%). The vaccination program ran in parallel with the EU average until the availability of the vaccines determined the vaccination rate.

However, from the point at which there were enough vaccines, since the speed of the vaccination program depended on population willingness and organization, which was low, vaccination coverage in Romania increasingly lagged behind the EU average. The vaccination program was hampered by communication problems and organizational problems, such as the late involvement of GPs and rural communities. Another problem with the organization of the program was that, according to an ECDC report, the Romanian government did not have information in November 2021 on the percentage of the population who were not yet vaccinated but were willing, uncertain, or not willing to get vaccinated [44]. The over-regulation of vaccination centers has also made it problematic for GPs to get involved in the vaccination program, as they have had difficulties complying with regulations. At the beginning of the fourth wave of the pandemic, the population vaccination rate was low, and the government applied less stringent restrictions, so hospitals were put under more severe pressure than in previous waves.

At the peak of the fourth wave of the pandemic, 24% more patients were admitted to the intensive care unit (99.4 patients per 1 million population) than at the peak of the third wave of the pandemic (80 patients per 1 million population) in the spring (Figure 2). However, due to the severe pressure on the health care system, several health services have been suspended, and Romania has requested medical assistance from the European Union by the Civil Protection Mechanism [42,45]. Moreover, it is noteworthy that the suspension of health services was applied during the third and fourth waves in 2021 and the first and second waves of pandemics in 2020, so the access to health services in Romania was severely limited for several periods [46].

Even before the COVID-19 pandemic, skepticism about vaccinations was a problem in Romania. According to Dascalu’s analysis in 2019, the control of the measles epidemic was hampered by low confidence in the health system, an insufficient healthcare workforce and resources to implement vaccination programs, ineffective communication of public health measures, and widespread anti-vaccination views in Romanian society [47]. As a result, a 2021 survey found that Romania had one of the highest rates of vaccine rejection (44%) among European countries [48]. In addition to these problems, the implementation of the COVID-19 vaccination program was hindered by the vaccine hesitancy among health care workers and the spread of anti-vaccination views by some members of the Romanian Orthodox Church [49,50,51]. Anti-vaccination and anti-restriction views were also widespread among political parties and their representatives [52,53]. Although the vaccination program has encountered many difficulties, several measures have been introduced in Romania since 2020 that have facilitated access to health services in 2021—such as telephone/online consultation and electronic document transmission [12]. In some cases, GPs also prescribed medication or care for chronic patients based on a written recommendation from a specialist.

### 3.4. Governance

In Romania, the National Emergency Management System (NEMS), established in 2004, has been used to prevent and manage various emergencies [54]. The central government operates the system and is directed by the National Committee for Special Emergency Situations. Several ministries, governmental organizations, and national professional organizations are also involved in the operation of NEMS. At the beginning of the pandemic, an advisory board of public health organizations was set up to provide professional support to NEMS [12]. In addition, the National Centre for Surveillance and Control of Communicable Diseases is responsible for the identification, surveillance and control of communicable diseases. Although the pandemic was managed primarily by the central government, measures could also be taken at the county level. A system was developed in 2020 that defined scenarios and associated restrictions based on the 14-day notification rate of newly reported COVID-19 cases per 100,000 population [55]. If the daily incidence rate reached 150 new cases per 100,000 people in a county, moderate restrictions, such as the closure of restaurants, cultural institutions, and entertainment venues, could have come into effect.

However, as the daily incidence rate reached 300 new cases per 100,000 people, severe restrictions, such as school closures and curfew restrictions, could occur. Although the system provided an opportunity for county decision-makers in 2020 and 2021 to apply local measures, this was not used uniformly, and in some cases, the central government did not apply the restrictions attached to the indicator for political or economic reasons [56]. In Romania, local elections were held on 27 September 2020, and parliamentary elections on 6 December 2020 [57]. According to OECD data, Romania recorded a notable decline in quarterly GDP in the second, third, and fourth quarters of 2020. However, except for Q1 2021, Romania’s quarterly GDP has already grown in 2021 [58]. The unpredictability of pandemic management may have also adversely affected the population’s willingness to comply. Consistent government action has been hampered by the fact that there have been several changes in government since the beginning of the pandemic, and by the end of 2021, six different Ministers oversaw the Ministry of Health [59].

### 3.5. Health Outcomes of Pandemic Management Measures to Slow down the Spread of the Pandemic

**Figure 3:**
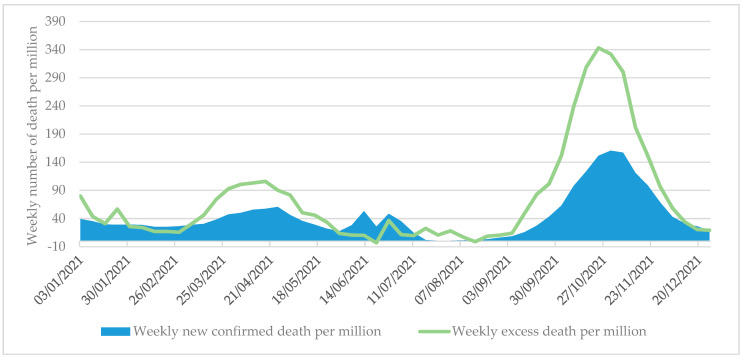
Weekly number of confirmed COVID-19 death per million and weekly excess death per million in Romania between 27 December 2020 and 26 December 2021. Sources: University of Oxford [21].

In Romania, significant COVID-19 related mortality was recorded in 2021 during the third and fourth waves; however, there was a discrepancy between the weekly confirmed mortality and the weekly excess mortality data (Figure 3).

While the cumulative number of confirmed deaths per million was 2265 from 27 December 2020 to 26 December 2021, the cumulative excess death per million was 3839. The difference between the two indicators was most substantial in the peak periods of the third and fourth waves. The reason behind the discrepancy is that weekly excess deaths may include (non-COVID-19-related) deaths that did not receive adequate care due to the severe pressure of the health care system. From the beginning of the pandemic in the spring of 2020 to 26 December 2021, there were 5616 excess deaths per million people, 68% of which occurred in 2021. Excess mortality in 2021 was more substantial than in 2020, despite the availability of vaccines and a wealth of evidence on the effectiveness of non-pharmaceutical measures. As of 26 December 2021, Romania had the third-highest excess mortality rate (5616 per million people) among the European Union Member States, with only Bulgaria and Lithuania showing a higher value (see Supplementary Table S3). Therefore, the Romanian government’s measures to manage the pandemic were less successful than in the other EU Member States, resulting in significant loss of life, a portion of which could have been avoided.

## 4. Discussion

Although vaccinations have given governments a new tool to combat COVID-19 in 2021, several studies have predicted that non-pharmaceutical measures may be needed in the early stages of vaccination programs and beyond [60,61]. Several studies have shown that the effectiveness of vaccinations is decreasing over time [62,63,64]. Therefore, governments had to speed up their vaccination programs and launch booster vaccination programs. In addition, if the vaccination coverage of the population was not adequate, governments had to take non-pharmaceutical measures appropriate to the epidemic to avoid health losses.

In Romania, the government did not apply extensive restrictions during the fourth wave of pandemic, despite a significant slowdown in the vaccination program from June 2021 onwards. Furthermore, the government did not make significant efforts on time to increase vaccination coverage. The combined effect of these actions (or lack of them) probably contributed to the loss of life in Romania during 2021. As in Romania, problems and challenges in many low- and middle-income countries have been caused by inadequate surveillance systems, limited access to vaccines, and false news about vaccinations [21,22,24]. However, some low- and middle-income countries have developed several good practices. For example, in Vietnam, a multi-sectoral response plan, comprehensive surveillance and contact research, transparent and wide-ranging communication, and uniform guidelines were used to successfully combat the pandemic [65].

In order to improve vaccination willingness, it is necessary to conduct a communication campaign developed for the needs of different social groups and improve access to the vaccination program [66]. A comprehensive testing strategy, a well-functioning surveillance system and an innovative contact research system are also needed to improve data quality and reduce delays [67].

Before the COVID-19 pandemic, the Romanian healthcare system faced severe problems due to outdated infrastructure and an inadequately structured workforce capacity. In the initial phase of the pandemic, these shortcomings, made worse by inadequate management, lack of protective equipment and safety protocols, and the dismissal of some health workers, which received extensive attention from the broader society [3]. After the initial phase, the Romanian government did implement several measures to provide protective equipment and educate the healthcare workforce in response to the crisis. According to a 2021 study by Kuhlmann et al., hospitals had adequate personal protective equipment (PPE) supplies, but long-term care facilities and primary care providers faced shortages [45]. Several factors influenced the suboptimal results of the COVID-19 vaccination program in Romania. Appropriate planning and provision of health infrastructure and workforce require the allocation of adequate financial resources and the flexible adaptation of regulations to the epidemic [68,69].

In addition to the factors mentioned in our analysis, the lack of adequate regulation of the sanctions for misinformation and the fact that even reliable news sites provided a broad platform for anti-vaccination perspectives can be identified as contributing factors [30]. Before the pandemic, a system was set up to manage public health emergencies in Romania. This system was put into operation when the pandemic broke out, with several additional health organizations invited to join. However, government pandemic management was inconsistent in 2020 and 2021 due to government instability, as well as low levels of public trust, coupled by reduced willingness to enact unpopular lockdowns close to parliamentary election [49]. Government instability and low trust in government have also made it difficult for other low- and middle-income countries to manage a pandemic [70]. According to the analysis of Badman and colleagues, trust in national and local public health institutions needs to be developed to increase the population’s willingness to cooperate with non-pharmaceutical measures. Public trust can be substantially increased through transparent, feedback-based, and clear communication [71].

According to several studies, access to health services in Romania was also disrupted in several phases of 2020 and 2021, which may have contributed to an increase in health losses [72,73]. According to the results of our study, in 2021, Romania had one of the highest cumulative excess mortality rates in the European Union. Romania already had the third-highest age-standardized cumulative excess mortality in the European Union in 2020, so it seems the Romanian government was unable to improve its pandemic management capabilities in 2021 compared to the previous year [74].

This study is subject to some limitations. Data on the daily confirmed COVID-19 cases may have been significantly affected by testing capacity; real case numbers could be under-detected at low testing capacity. At the peaks of the epidemic waves, limited testing capacity and data collection difficulties may have negatively affected the quality of data on hospitalization and mortality, under-estimating actual case numbers. The use of the stringency index also has some limitations. The data are updated to the database on twice-weekly cycles, but not every country is updated in every cycle, and missing or not fully complete data also occur. The stringency index can only be interpreted by examining several other indicators together. However, the index can be used to characterize restrictive measures by governments because it provides a comprehensive picture on the nature of the most significant measures. The OWID database’s limitation is that some indicators are not updated in every country in each cycle and missing or not fully complete data may also occur. However, for Romania, the database does not contain incomplete data for the examined indicators for 2021.

## 5. Conclusions

Our analysis of the management of the 2021 pandemic in Romania illustrates several experiences. In 2021, non-pharmaceutical measures were not applied in a timely and adequate manner in Romania. Although health infrastructure and workforce have been developed compared to 2020, institutions have been under severe pressure at the peak of the third and fourth wave of pandemics. Due to the shortcomings in implementing the vaccination program, vaccination coverage remained low, so both COVID-19 and non-COVID-19 patients had limited access to health services during the fourth wave of the pandemic.

Based on our analysis, several lessons can be learned. In order to tackle an epidemic such as COVID-19 with success at a national level, several key factors have to be in place. First and foremost, a complex monitoring and management system and a stable government that enjoys the trust of the public. Valid information on the spread of the epidemic can only be provided with extensive testing capacity. In addition, the variant monitoring system allows identifying and tracking a new variant of concern. Innovative monitoring capabilities make it possible to provide a wide range of information, making decision-makers more likely to take appropriate action to the risks. With the help of these monitoring systems, a decrease in the effectiveness of vaccinations over time and against new variants can also be monitored. Creating and enforcing legislation that can reduce the spread of false information is also a key factor. With appropriate management systems and mechanisms, government action can be taken promptly to address risks identified at an early stage. The healthcare system can be prepared for the pressure caused by the pandemic wave, and thus, the burnout of health care workers or the suspension of services can be prevented.

## Figures and Tables

**Table 1:** Indicators used in the analysis and their sources.

Indicator	Sources
Confirmed COVID-19 cases per million	University of Oxford
Weekly test positivity rate	European Centre for Disease Control and Prevention
Stringency index	University of Oxford
Practicing medical doctors, medical graduates, and available hospital beds per hundred thousand inhabitants	European Centre for Disease Control and Prevention
Number of COVID-19 patients in ICU per million	University of Oxford
Number of fully vaccinated people per hundred	University of Oxford
Share of fully vaccinated people by age groups	European Centre for Disease Control and Prevention
Weekly number of confirmed COVID-19 death per million	University of Oxford
Weekly excess death per million	Our World in Data

Sources: Authors own elaborations.

**Table 2:** Most important non-pharmaceutical measures in Romania between 1 January 2021 and 31 December.

Date	Measures	Sources
In force on 1 January 2021	Mandatory mask wearing in indoor and outdoor public spaces, stay-at home requirement between 23:00 and 05:00, school closure, closure of markets in closed spaces, restaurants and cafes could operate at reduced capacity and opening hours, restrictions on private gatherings, mandatory remote working where possible.	[32]
8 February 2021	Opening of kindergartens and primary schools, partial opening of secondary schools based on epidemic situation	[33]
8 March 2021	Stay-at home restrictions between 22:00 and 05:00, restaurants and cafes operate at reduced opening hours	[34]
2 April 2021	Extended holiday in schools until 4 May 2021 to reduce mobility	[33]
15 May 2021	Removal of stay-at home requirements, release mandatory mask wearing on outdoor spaces, partial release ban on mass gatherings, reopen cultural centers	[35]
1 June 2021	Lifting restrictions on the attendance of vaccinated people at concerts, weddings, cultural and religious events; reopen gyms and sport centres, mass gatherings up to 1000 people are permitted	[36]
10 August 2021	Gatherings up to 10 people are permitted, mandatory mask wearing in crowded spaces, social distancing and capacity restrictions for busnisses	[37]
25 October 2021	Stay-at home restrictions between 22:00 and 05:00 except for vaccinated and recently recovered; health passes and reduced opening hours for busnisses and restaurants	[38]

Sources: Authors own elaborations.

**Table 3:** Practicing medical doctors, medical graduates, and available hospital beds per hundred thousand inhabitants in Romania and the EU 27 average in 2019.

	Romania	EU 27 Countries (Average)
Practising medical doctors	318.6	390.5
Medical graduates	25.6	14.3
Hospital beds	705.7	531.9

Sources: Eurostat [23].

## Data Availability

Publicly available datasets were analyzed in this study. These data can be found here: https://ourworldindata.org/explorers/coronavirus-data-explorer (accessed on 3 March 2022); https://www.ecdc.europa.eu/en/covid-19/data (accessed on 3 March 2022); https://ec.europa.eu/eurostat/data/database (accessed on 3 March 2022); https://data.oecd.org/gdp/quarterly-gdp.htm (accessed on 3 March 2022). The data underlying this article will be shared on reasonable request to the corresponding author.

## Supplementary Materials

The following are available online at https://www.mdpi.com/article/10.3390/tropicalmed7070122/s1, Figure S1: Weekly test positivity rate in Romania between 1 January and 31 December 2021. Source: ECDC (2022), Table S1: Sequencing volume sufficient to estimate variant proportions at 5% prevalence in Romania in 2021 between Week 1 and Week 52, Table S2: Share of fully vaccinated people by age groups in Romania and EU Member States in 2021 Week 35 and Week 52, Table S3: Cumulative excess mortality per million people in the EU Member States as of 26 December 2021.
