# Supplementary material for: Riding the Pandemic Waves—Lessons to Be Learned from the COVID-19 Crisis Management in Romania
Source: Trop Med Infect Dis. 2022 Jun 29;7(7):122. doi: 10.3390/tropicalmed7070122 (PMC9316926; doi:10.3390/tropicalmed7070122)
Supplement: Supplementary file 1 [file tropicalmed-07-00122-s001.zip › tropicalmed-1769513-supplementary.pdf]

Tropical Medicine and Infectious Disease

MDPI

Supplementary Materials: Riding the Pandemic Waves—Lessons to Be Learned from the COVID-19 Crisis Management in Romania

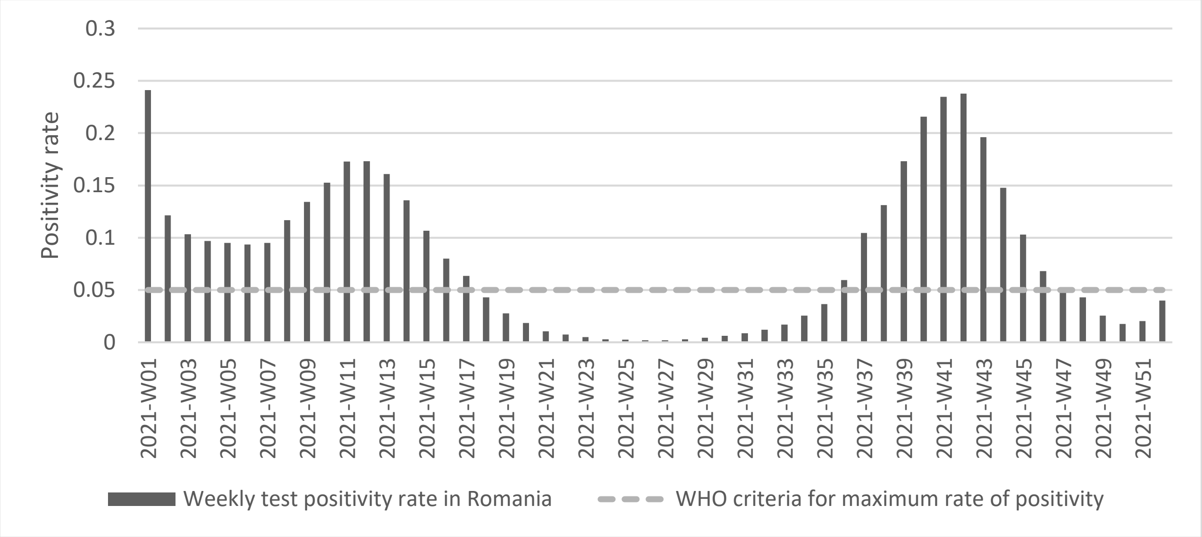

Figure S1. Weekly test positivity rate in Romania between 1 January and 31 December 2021. Source: ECDC (2022).

Table S1. Sequencing volume sufficient to estimate variant proportions at 5% prevalence in Romania in 2021 between Week 1 and Week 52.

Year Week	Weekly New cases	Number Sequenced Reported to GISAID	Percent Cases Sequenced Reported to GISAID	Number Sequenced Reported to TESSY	Percent Cases Sequenced Reported to TESSY	Sequencing Volume Sufficient to Estimate Variant Proportions at 5% Prevalence
2021-01	29,409	69	0.2	68	0.2	Insufficient
2021-02	20,757	47	0.2	0	0	Insufficient
2021-03	17,383	53	0.3	14	0.1	Insufficient
2021-04	17,240	71	0.4	132	0.8	Insufficient
2021-05	16,659	100	0.6	105	0.6	Insufficient
2021-06	16,498	67	0.4	82	0.5	Insufficient
2021-07	18,404	101	0.5	90	0.5	Insufficient
2021-08	23,068	142	0.6	45	0.2	Insufficient
2021-09	27,212	128	0.5	228	0.8	Insufficient
2021-10	32,564	180	0.6	77	0.2	Insufficient
2021-11	38,123	88	0.2	185	0.5	Insufficient
2021-12	39,497	121	0.3	16	0	Insufficient
2021-13	37,268	183	0.5	128	0.3	Insufficient
2021-14	30,084	119	0.4	195	0.6	Insufficient
2021-15	22,125	74	0.3	94	0.4	Insufficient
2021-16	16,108	94	0.6	34	0.2	Insufficient
2021-17	10,403	39	0.4	154	1.5	Insufficient
2021-18	8313	117	1.4	101	1.2	Insufficient
2021-19	5399	50	0.9	63	1.2	Insufficient
2021-20	3389	70	2.1	141	4.2	Insufficient
2021-21	1922	82	4.3	25	1.3	Insufficient
2021-22	1224	61	5	528	43.1	Sufficient
2021-23	841	58	6.9	390	46.4	Sufficient
2021-24	476	49	10.3	208	43.7	Sufficient

2021-25	393	23	5.9	386	98.2	Sufficient
2021-26	298	32	10.7	100	33.6	Insufficient
2021-27	305	26	8.5	190	62.3	Sufficient
2021-28	431	29	6.7	110	25.5	Insufficient
2021-29	703	68	9.7	148	21.1	Insufficient
2021-30	1138	110	9.7	330	29	Sufficient
2021-31	1645	152	9.2	170	10.3	Insufficient
2021-32	2536	148	5.8	98	3.9	Insufficient
2021-33	3891	133	3.4	192	4.9	Insufficient
2021-34	6315	234	3.7	350	5.5	Sufficient
2021-35	9866	355	3.6	386	3.9	Sufficient
2021-36	16,541	506	3.1	700	4.2	Sufficient
2021-37	30,867	553	1.8	758	2.5	Sufficient
2021-38	48,182	501	1	954	2	Sufficient
2021-39	76,707	488	0.6	1038	1.4	Sufficient
2021-40	91,906	589	0.6	702	0.8	Sufficient
2021-41	101,812	304	0.3	702	0.7	Sufficient
2021-42	103,649	372	0.4	940	0.9	Sufficient
2021-43	81,597	264	0.3	572	0.7	Sufficient
2021-44	54,463	158	0.3	510	0.9	Sufficient
2021-45	31,806	111	0.3	666	2.1	Sufficient
2021-46	18,951	155	0.8	384	2	Sufficient
2021-47	12,882	65	0.5	546	4.2	Sufficient
2021-48	8201	77	0.9	330	4	Sufficient
2021-49	6335	76	1.2	222	3.5	Insufficient
2021-50	4686	175	3.7	374	8	Sufficient
2021-51	4424	78	1.8	334	7.5	Sufficient
2021-52	9351	135	1.4	778	8.3	Sufficient

Sources: ECDC (2022).

Table S2. Share of fully vaccinated people by age groups in Romania and EU Member States in 2021 Week 35 and Week 52.

Week	Age Group	Romania	EU
**2021 W 35**	18-24 years	25.7%	49.2%
	25-49 years	30.7%	60.1%
	50-59 years	37.3%	72.5%
	60 + years	34.3%	82.9%
**2021 W 52**	18-24 years	47.2%	71.3%
	25-49 years	48.2%	74.9%
	50-59 years	55.2%	81.0%
	60 + years	45.5%	89.1%

Sources: ECDC (2022).

Table S3. Cumulative excess mortality per million people in the EU Member States as of December 26. 2021.

Country	Excess Death per Million
Bulgaria	8344
Lithuania	5917
Romania	5616
Slovakia	4444
Poland	4067
Croatia	4021
Czechia	3838
Latvia	3690
Hungary	3660
Italy	2768
Slovenia	2362
Estonia	2349

Greece	2288
Spain	2186
Portugal	2039
Belgium	1755
Austria	1679
Netherlands	1637
France	1153
Germany	1025
Sweden	964
Cyprus	843
Malta	789
Ireland	593
Finland	480
Denmark	136
Luxembourg	83

Sources: University of Oxford (2022).
